# Assessment of Pain, Diet, and Analgesic Use in Orthodontic Patients: An Observational Study

**DOI:** 10.3390/medicina61020357

**Published:** 2025-02-19

**Authors:** Bianca Maria Negruțiu, Claudia Elena Staniș, Ligia Luminița Vaida, Abel Emanuel Moca, Cristina Paula Costea, Raluca Iurcov, Alexandru Nicolae Pîrvan, Marius Rus

**Affiliations:** 1 Department of Dental Medicine, Faculty of Medicine and Pharmacy, University of Oradea, 10 Piața 1 Decembrie Street, 410073 Oradea, Romanialigia_vaida@uoradea.ro (L.L.V.); cristinacostea@uoradea.ro (C.P.C.); 2 Department of Medical Disciplines, Faculty of Medicine and Pharmacy, University of Oradea, 10 Piața 1 Decembrie Street, 410073 Oradea, Romaniarusmarius@uoradea.ro (M.R.); 3 Department of Surgical Disciplines, Faculty of Medicine and Pharmacy, University of Oradea, 10 Piața 1 Decembrie Street, 410073 Oradea, Romania; alexandrupirvan88@gmail.com

**Keywords:** orthodontics, pain, oral health, gender, age, diet

## Abstract

*Background/Objectives*: Orthodontic treatment offers significant functional and aesthetic benefits, but it often causes discomfort, impacting patients’ daily activities, including diet and medication use. The primary aim of this observational study was to assess the influence of orthodontic patients’ age, gender, and living environment on pain duration, diet impairment, and analgesic use one week after their first adjustment visit to the orthodontist. *Methods*: This observational study included a sample of 194 orthodontic patients who completed a questionnaire consisting of six single-choice questions. The questions addressed the following variables: age, gender, living environment, pain duration, diet impairment, and analgesic use. The main inclusion criteria encompassed undergoing fixed orthodontic treatment on the upper arch for one week using a 0.0016-inch superelastic NiTi archwire (American Orthodontics, Sheboygan, WI, USA) and presenting moderate to severe crowding of the upper and lower anterior teeth. *Results*: Female patients were significantly more likely to experience diet impairment than males (51.9% vs. 33.3%, *p* = 0.013). Among female patients reporting pain lasting approximately one week, a higher proportion resided in rural areas (25% vs. 6.8%, *p* = 0.045), whereas male patients reporting the same pain duration predominantly lived in urban areas (12.5% vs. 0%, *p* = 0.028). Overall, 55.7% of participants used analgesics, with females in rural areas being the most frequent users. Additionally, patients who used analgesics were significantly more likely to experience diet impairment than those who did not (64.3% vs. 49.1%, *p* = 0.041). *Conclusions*: Female patients residing in rural areas reported a longer duration of pain, which led to a greater likelihood of experiencing diet impairment and increased consumption of analgesics compared to their counterparts. These findings highlight the need for personalized pain management strategies in orthodontic treatment, especially for patients with increased vulnerability to discomfort.

## 1. Introduction

Undergoing orthodontic treatment offers several benefits, including improved aesthetics, enhanced occlusal function, and increased self-esteem and self-confidence. However, it may also cause discomfort as a response to both physical and psychological factors [1,2].

Orthodontic patients often experience significant anxiety and stress, not only during bonding but also during adjustment visits, which can negatively impact their social interactions and self-confidence [3]. To mitigate these psychological effects, orthodontists must implement strategies to effectively address stress and anxiety. Malocclusion, particularly when accompanied by facial irregularities, has a profound effect on self-esteem and confidence [4]. For instance, conditions such as deep bite and crowding have been shown to significantly diminish facial attractiveness, impairing social interactions and exacerbating anxiety [5]. Conversely, well-aligned teeth can significantly enhance self-perception and social confidence [6].

Additionally, a substantial proportion of orthodontic patients (91–95%) report experiencing pain at various stages of treatment, rather than mere discomfort [7]. This pain often adversely affects patients’ attitudes toward their orthodontic care, leading to reduced quality of life, diminished satisfaction, and decreased self-esteem. These outcomes, in turn, contribute to poor adherence to treatment protocols [8,9,10,11,12], compromised oral hygiene, and indifference to treatment progress [13]. In cases involving extractions, tooth movement exerts pressure on the attached gingiva, causing pain and bleeding during brushing, which often results in patients ceasing oral hygiene practices and delaying treatment [13]. Although the mechanisms underlying orthodontic discomfort are not fully understood, it is believed to involve inflammatory, vascular, cellular, immunological, and neural reactions triggered by orthodontic forces [14]. To alleviate pain and minimize bracket breakage, orthodontists frequently advise patients to avoid hard and sticky foods, leading to altered dietary habits and, in some cases, weight loss during treatment [15].

The perception of orthodontic pain can be influenced by various factors. While several studies have concluded that age and gender do not significantly impact pain levels [16,17,18,19], emerging evidence suggests that these variables may influence patients’ experiences of orthodontic pain [20,21].

Limited research has explored the impact of gender and living environment on orthodontic pain, particularly in Eastern European settings. This article aims to address these gaps and provide insights for improved pain management.

The aim of this observational study was to assess the correlation between pain duration, analgesic use, and diet impairment, considering patients’ gender and environment of origin, in individuals undergoing fixed orthodontic treatment for one week following the initial placement of an archwire. The primary objective was to evaluate the influence of age, gender, and environment of origin on pain duration, diet impairment, and analgesic use during the first week of orthodontic treatment.

This study tested the following null hypotheses:

- There is a significant association between demographic factors and orthodontic pain.
- There is a significant association between demographic factors and diet impairment in orthodontic patients.
- There is a significant association between demographic factors and analgesic use in orthodontic patients.

## 2. Materials and Methods

### 2.1. Ethical Considerations

This study was conducted in accordance with the ethical principles outlined in the 1964 Declaration of Helsinki and its subsequent amendments. Ethical approval was granted by the Research Ethics Committee of the University of Oradea (Approval No. 23/09 June 2023). Informed consent was obtained from all participants or their legal guardians prior to their inclusion in this study. Prior to completing the questionnaire, respondents were informed that participation was entirely voluntary and anonymous, with no financial or other incentives provided.

### 2.2. Participants and Data Collection

This observational study was conducted from July 2023 to May 2024 on a group of orthodontic patients from Oradea, Romania. Participants were administered a paper-based questionnaire, written in Romanian, which was easy to understand and complete. The questionnaire consisted of six items investigating the following aspects: age, gender, living environment, pain duration, diet impairment, and administration of painkillers (Table 1). The questionnaire was distributed on the seventh day of orthodontic treatment during a routine visit to the specialist.

The questionnaire was completed by the patient or, in the case of minors, by their legal guardian, while waiting in the clinic’s reception area prior to their consultation.

Before distribution, the questionnaire underwent a validation process to ensure its reliability and appropriateness for measuring the constructs of interest, including pain duration, diet impairment, and analgesic use. First, five specialists in orthodontics reviewed the questions to assess content validity, ensuring alignment with the study objectives and clinical relevance. Minor revisions were made to improve clarity and specificity. Subsequently, the revised survey was piloted with 20 orthodontic patients, meeting the inclusion criteria. Feedback on question comprehension and ease of response was collected, leading to further adjustments to enhance readability. Internal consistency was evaluated using the pilot data, and Cronbach’s alpha coefficient for the six survey items was calculated, yielding a value of 0.81, indicating good reliability. This result demonstrates that the questions were consistent in measuring the intended constructs and provided robust data for this study.

The inclusion criteria for this study required participants to be undergoing fixed orthodontic treatment on the upper arch for one week and to present with moderate to severe crowding of the upper and lower anterior teeth. Eligible participants were non-syndromic, with no craniofacial deformities or cleft lips or palates, and had no history of previous orthodontic treatment. Additionally, participants had to be free from systemic diseases or medications that could influence pain perception, such as autoimmune diseases (e.g., rheumatoid arthritis, systemic lupus erythematosus), arthritis, joint or bone disorders, or acute gout.

Exclusion criteria encompassed patients with complex orthodontic cases, such as malocclusions requiring extractions or those necessitating orthognathic surgery. Patients were also excluded if they had known allergies to nickel or other materials used in orthodontic appliances. Other exclusion factors included chronic pain conditions, orofacial pain unrelated to orthodontic treatment, active caries, or periodontal disease at the time of treatment. Additionally, patients with systemic health conditions that could influence pain perception or interfere with treatment outcomes were not eligible for inclusion. To ensure a homogenous study group, patients with known gnathological alterations, such as temporomandibular disorders (TMDs) or occlusal dysfunctions, were also excluded.

For all participants, the initial treatment involved 0.022-inch slot Roth prescription brackets (American Orthodontics, Sheboygan, WI, USA) on the upper arch. The initial archwire used was a 0.0016-inch superelastic NiTi wire (American Orthodontics, Sheboygan, WI, USA).

### 2.3. Statistical Analysis

All data collected in this study were analyzed using IBM SPSS Statistics 25 (IBM Corp., Armonk, NY, USA) with visual representations created in Microsoft Office Excel and Word 2024 (Microsoft Corp., Redmond, WA, USA). Qualitative variables were summarized as counts and percentages, and comparisons between groups were conducted using Fisher’s exact test. Univariate binomial logistic regression models were employed to identify significant correlations observed in the contingency tables. These models were evaluated for significance and goodness-of-fit. Correlations determined by the models were expressed as odds ratios with 95% confidence intervals, accompanied by corresponding significance values. A threshold significance level of α = 0.05 was applied for all statistical tests.

The required sample size was determined using G*Power 3.1.9.7 software (Heinrich-Heine-Universität Düsseldorf, Düsseldorf, Germany). A power analysis for a logistic regression model (two-tailed, α = 0.05, power = 0.80, medium effect size, OR = 1.68, df = 1) indicated that a minimum of 150 participants was needed. The final sample of 194 patients ensured adequate statistical power to detect significant associations.

## 3. Results

**Figure 1:**
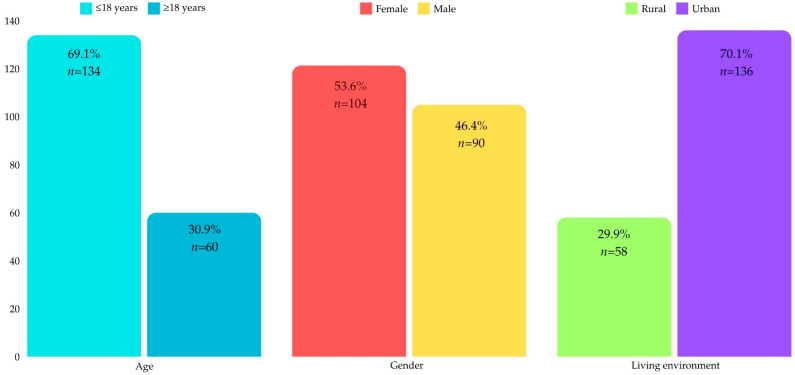
Sample characteristics.

Following the application of the inclusion and exclusion criteria, the final sample consisted of 194 orthodontic patients. According to the age distribution of the patients, 2 individuals (1%) were between 6 and 12 years of age, 132 individuals (68%) were aged 13 to 18 years, and 60 individuals (30.9%) were over 18 years old. Regarding gender, 104 patients (53.6%) were female, while 90 patients (46.4%) were male. In terms of living environment, 58 patients (29.9%) were from rural areas, whereas the majority, 136 patients (70.1%), resided in urban areas (Figure 1).

Table 1 presents the distribution of patients according to age, gender, and pain duration. The majority of patients, 178 (91.8%), reported experiencing pain for several days, while 16 patients (8.2%) reported pain lasting one week. No significant associations were observed between pain duration and gender (*p* = 0.603) or age (*p* = 0.780). Similarly, no significant differences in pain duration were identified when analyzing female patients by age (*p* = 0.749) or male patients by age (*p* = 1.000).

Gender, pain duration, and diet impairment were analyzed to understand their interrelationships. Among the patients, 84 (43.3%) reported experiencing diet impairment. This condition showed no significant association with pain duration (*p* = 0.794), a result consistent when examining female patients by pain duration (*p* = 0.743) and male patients by pain duration (*p* = 0.173). However, diet impairment was significantly more prevalent among female patients (51.9%) compared to males (33.3%) (*p* = 0.013). A binomial logistic regression analysis further indicated that female patients had 2.160 times higher odds of experiencing diet impairment (95% CI: 1.206–3.870, *p* = 0.010) than male patients (Table 2).

The distribution of patients based on gender, living environment, and pain duration revealed notable trends. Among the study group, males were predominantly from rural areas (46.7% vs. 15.4%), whereas females were more commonly from urban areas (84.6% vs. 53.3%) (*p* < 0.001). Across the entire study sample, pain duration was not significantly associated with the living environment (*p* = 0.781). However, gender-specific analysis showed distinct patterns. In the female subgroup, a significantly higher proportion of patients who experienced pain for about a week were from rural areas (25% vs. 6.8%, *p* = 0.045). Conversely, in the male subgroup, most patients reporting pain for about a week were from urban areas (12.5% vs. 0%, *p* = 0.028).

Due to data limitations, the correlation between living environment and pain duration in male patients could not be quantified using logistic regression models. In the female subgroup, however, patients from rural areas had 4.556 times higher odds of experiencing pain lasting about a week compared to those from urban areas (95% CI: 1.120–18.524, *p* = 0.034). This association remained significant even after adjusting for age in a multivariable logistic regression model (rural background: OR = 5.123, 95% CI: 1.202–21.836, *p* = 0.027; age > 18 years: OR = 0.583, 95% CI: 0.143–2.379, *p* = 0.452) (Table 3).

Table 4 presents the distribution of patients according to gender, living environment, and analgesic use. Of the 194 patients included in this study, 108 (55.7%) reported using analgesics to alleviate pain. Female patients were found to use analgesics significantly more frequently than male patients (63.5% vs. 46.7%, *p* = 0.021). Across the entire study sample, the use of analgesics was not significantly associated with the living environment (*p* = 0.083). However, gender-specific analysis revealed that in the female subgroup, analgesic use was significantly more prevalent among those living in rural areas compared to urban areas (87.5% vs. 59.1%, *p* = 0.046), whereas no statistically significant correlation was observed in the male subgroup (*p* = 0.090).

Binomial logistic regression analysis indicated that patients residing in rural areas had 4.846 times higher odds of using analgesics compared to those from urban areas (95% CI: 1.038–22.635, *p* = 0.045). This association remained significant in a multivariable logistic regression model adjusted for age (rural background: OR = 5.066, 95% CI: 1.072–23.941, *p* = 0.041; age > 18 years: OR = 0.807, 95% CI: 0.353–1.845, *p* = 0.612).

Table 5 presents the distribution of patients based on gender, diet impairment, and analgesic use. Within the entire study group, patients who used analgesics were significantly more likely to experience diet impairment than those who did not (64.3% vs. 49.1%, *p* = 0.041). Gender-specific analysis revealed that this association persisted in the female subgroup, where patients using analgesics more frequently reported diet impairment (77.8% vs. 48%, *p* = 0.002). However, in the male subgroup, the differences were not statistically significant (*p* = 0.502).

## 4. Discussion

In recent years, a growing number of patients have sought orthodontic treatment due to increased self-awareness and the pervasive influence of social media. Digital technology can negatively impact individuals’ mental well-being and self-esteem, amplifying the desire for orthodontic care [4]. Given that orthodontic treatments are often long-lasting and may involve discomfort or pain [3], it is essential for orthodontists to foster open, honest, and trusting relationships with their patients. To improve patients’ attitudes toward orthodontic treatment, clinicians can utilize tools such as initial photographs, radiographs, and dental models during adjustment visits to demonstrate progress and set realistic expectations [22]. Additional strategies for improving patient compliance include establishing a strong family support system, maintaining transparent communication that emphasizes long-term benefits, and encouraging patients to learn from the practical advice of others who have undergone similar treatments [4].

The present study included 194 patients undergoing fixed orthodontic treatment on the upper arch for one week at three private practices located in the northwestern region of Romania, specifically in Oradea, Bihor County. The study group was stratified by gender (104 females and 90 males), age (134 patients under the age of 18 and 60 patients over 18), and environment of origin (136 patients living in urban areas represented by the city of Oradea and 58 patients living in rural areas of Bihor County). The age distribution revealed that 2 patients (1%) were aged between 6 and 12 years, 132 patients (68%) were aged between 13 and 18 years, and 60 patients (30.9%) were over 18 years. Among the participants, 104 patients (53.6%) were female, and 90 patients (46.4%) were male. Most of the patients (136; 70.1%) resided in urban areas, while 58 patients (29.9%) lived in rural areas.

According to our findings, the majority of patients diagnosed with moderate to severe crowding of the upper and lower anterior teeth reported pain lasting several days, but not one week, regardless of gender, age, or environment of origin, following the first week of orthodontic treatment. This high prevalence of reported pain can be attributed to the leveling and aligning phase of treatment, particularly when crowding affects the anterior teeth, which have smaller root surfaces and are more frequently used for biting [23]. Additionally, Inauen DS et al. (2023) suggested that pain induced by orthodontic appliances during chewing, biting, or intercuspation is more pronounced than spontaneous pain, as masticatory muscles exert a compressive effect on the sensitized periodontal ligament [24]. Our findings align with those of Chan V. et al. (2024), who reported that pain associated with orthodontic fixed therapy peaked two days after an adjustment appointment and remained elevated for seven days, particularly with the introduction of new archwire materials [25,26]. Similarly, Wei Lin et al. (2021) found that sex, age, and genetic polymorphisms had minimal or no influence on perceived pain levels [27].

Most patients in our study did not experience diet impairment, regardless of pain duration or gender. However, among the 43.3% of patients who did report diet impairment, females were significantly more affected than males, with females having 2.16 times greater odds of experiencing diet impairment. These results are consistent with those reported by Sultan H. et al. (2023) and Asiry Ma. et al. (2014), who also observed high rates of diet impairment among orthodontic patients [28,29].

Orthodontic patients often experience dietary restrictions because orthodontists typically recommend a soft diet to minimize pain and prevent appliance breakage, which can impact patients’ nutritional status [30]. Reducing appliance breakage not only enhances patient satisfaction but also improves the efficiency of appointments [31]. Recent studies have highlighted that such dietary changes can positively influence patients’ health, as they tend to adopt healthier eating habits, avoiding sticky foods such as chocolates and snacks [32,33]. However, Fang D et al. reported that inadequate nutrition in orthodontic patients adversely affects the biological response of the periodontal ligament and bone to orthodontic forces. To mitigate the risk of malnutrition during orthodontic treatment, collaboration between orthodontists and nutritionists is recommended to ensure a comprehensive approach to patient care [34].

Our findings reveal that more than 55% of the patients in the study group used analgesics, regardless of their living environment. Female patients were more likely to use analgesics compared to males, with the majority of female analgesic users residing in rural areas. In contrast, no statistically significant differences were observed among male patients. Patients living in rural areas were 4.846 times more likely to use analgesics compared to those in urban areas. These results align with findings by Sultan H et al. (2023), who reported that 54.6% of patients in their study group relied on painkillers after adjustment visits [28].

This study identified significant differences in pain perception, diet impairment, and analgesic use between urban and rural patients. Patients from rural areas reported higher levels of pain and greater reliance on analgesics, which may be attributed to several socio-environmental factors. One key factor is socioeconomic fragility. Rural populations often face challenges such as lower socioeconomic status, limited access to healthcare resources, and higher rates of health risk behaviors. These disparities can exacerbate health outcomes, including pain perception and management [35]. Additionally, dietary habits differ between urban and rural populations. Studies have shown that rural populations may consume diets with different consistencies, which can influence oral health and pain perception during orthodontic treatment [36]. Furthermore, mental health status and social support play crucial roles in pain perception. Rural residents may experience higher levels of psychological stress and have less access to social support networks, which can amplify pain experiences [37].

High levels of discomfort or pain following the adjustment of fixed orthodontic appliances can be attributed to the release of inflammatory mediators within the first 24–48 h due to the compression of the periodontal ligament. This discomfort is triggered by nociceptors in the periodontal ligament that become sensitized by these inflammatory mediators [38]. Additionally, the use of analgesics may influence the duration of orthodontic treatment, as some studies suggest that painkillers can alter the rate of tooth movement in animal models [39,40].

According to our study, patients who used analgesics to alleviate orthodontic pain were more likely to experience diet impairment compared to those who did not use painkillers. This trend was particularly pronounced among female patients, who were also more likely to report diet impairment after the first week of orthodontic treatment. In contrast, no statistically significant differences were observed among male patients. The heightened response to orthodontic stimuli observed in females may be explained by hormonal fluctuations during the menstrual cycle and the influence of ovarian steroids [20,41]. Similarly, Sultan H et al. (2023) reported that female patients experienced higher levels of pain than males following the insertion of separators [28]. Among female patients, those residing in rural areas were 4.556 times more likely to report pain lasting approximately one week compared to their urban counterparts, whereas male patients showed no significant geographic differences.

Orthodontic movement of endodontically treated teeth may be influenced by their altered biomechanical properties, as studies have shown that the mechanical performance of these teeth depends on the type of fiber-reinforced post and the bonding interface between dentin, post, and cement, which can also impact pain perception during orthodontic treatment [42].

The findings of this study support the research hypotheses, confirming that demographic factors such as gender and living environment are significantly associated with orthodontic pain perception, diet impairment, and analgesic use. Specifically, female patients and those from rural areas reported a higher prevalence of prolonged pain, greater dietary impairment, and more frequent analgesic use.

This study has certain limitations, including the complexity of malocclusions and the subjective nature of self-reported pain perception. Beyond psychosocial and biophysiological factors, pain can also be influenced by mental health status, socioeconomic conditions, and familial or sibling dynamics [43]. A control or reference group was not included, which limits the ability to establish causal relationships between orthodontic treatment and the assessed variables. However, this study provides valuable observational insights into pain duration, diet impairment, and analgesic use in orthodontic patients. Future studies should incorporate a control group for direct comparisons. Furthermore, while this study assessed analgesic use, it did not collect specific information on the types or frequency of analgesic medications taken. This limits the ability to evaluate the potential differences in pain perception and dietary impact between different classes of analgesics. Future research should include detailed analgesic usage patterns to provide a more comprehensive understanding of their role in managing orthodontic pain. Additionally, the sample was limited to a single geographic region (Oradea, Romania), which may affect generalizability. This study also focused only on the first week of treatment, without long-term follow-up. Finally, while the sample size was justified, larger studies incorporating psychological and socioeconomic factors would provide more comprehensive insights.

Future research should explore longitudinal changes in pain perception, dietary diversity, and weight loss throughout the entirety of orthodontic treatment, taking into account patients’ malocclusions and other influencing factors. The clinical significance of our findings lies in the improved management of resources such as patient motivation, education, and the use of both pharmacological and non-pharmacological interventions to alleviate orthodontic pain. These results can serve as a valuable guide for clinicians in effectively addressing orthodontic pain and enhancing patient care.

## 5. Conclusions

This study assessed the influence of gender, age, and living environment on pain perception, diet impairment, and analgesic use in orthodontic patients. The key findings are as follows:

- Most patients experienced pain for several days following the first week of orthodontic treatment, with no significant differences based on gender, age, or living environment.
- Female patients were more likely to experience diet impairment and use analgesics compared to males.
- Patients from rural areas, especially females, reported longer pain durations, which led to greater diet impairment and increased analgesic use.
- Analgesic use was significantly associated with diet impairment, particularly among female patients.
- The living environment influenced pain perception and analgesic use, suggesting that socio-environmental factors play a role in orthodontic pain experiences.
- These findings emphasize the need for personalized pain management strategies, particularly for vulnerable groups such as female patients in rural areas.

## Figures and Tables

**Table 1:** Distribution of the patients according to age, gender, and pain duration.

**Age**					
**Pain duration**	≤18 years		>18 years		*p* *
	No.	%	No.	%	
Several days	122	91%	56	93.3%	0.780
One week	12	9%	4	6.7%	
**Gender**					
**Pain duration**	Female		Male		*p* *
	No.	%	No.	%	
Several days	94	90.4%	84	93.3%	0.603
One week	10	9.6%	6	6.7%	
**Female respondents—Age**					
**Pain duration**	≤18 years		>18 years		*p* *
	No.	%	No.	%	
Several days	50	89.3%	44	91.7%	0.749
One week	6	10.7%	4	8.3%	
**Male respondents—Age**					
**Pain duration**	≤18 years		>18 years		*p* *
	No.	%	No.	%	
Several days	72	92.3%	12	100%	1.000
One week	6	7.7%	0	0%	

* Fisher’s exact test; No.—number.

**Table 2:** Distribution of the patients according to gender, pain duration, and diet impairment.

**Gender**					
**Diet impairment**	Female		Male		*p* *
	No.	%	No.	%	
Absent	50	48.1%	60	66.7%	0.013
Present	54	51.9%	30	33.3%	
**Pain duration**					
**Diet impairment**	Several days		One week		*p* *
	No.	%	No.	%	
Absent	100	56.2%	10	62.5%	0.794
Present	78	43.8%	6	37.5%	
**Pain duration—Female respondents**					
**Diet impairment**	Several days		One week		*p* *
	No.	%	No.	%	
Absent	46	48.9%	4	40%	0.743
Present	48	51.1%	6	60%	
**Pain duration—Male respondents**					
**Diet impairment**	Several days		One week		*p* *
	No.	%	No.	%	
Absent	54	64.3%	6	100%	0.173
Present	30	35.7%	0	0%	

* Fisher’s exact test; No.—number.

**Table 3:** Distribution of the patients according to gender, environment of origin, and pain duration.

**Gender**					
**Living environment**	Female		Male		*p* *
	No.	%	No.	%	
Rural	16	15.4%	42	46.7%	<0.001
Urban	88	84.6%	48	53.3%	
**Living environment**					
**Pain duration**	Rural		Urban		*p* *
	No.	%	No.	%	
Several days	54	93.1%	124	91.2%	0.781
One week	4	6.9%	12	8.8%	
**Living environment—Female respondents**					
**Pain duration**	Rural		Urban		*p* *
	No.	%	No.	%	
Several days	12	75%	82	93.2%	0.045
One week	4	25%	6	6.8%	
**Living environment—Male respondents**					
**Pain duration**	Rural		Urban		*p* *
	No.	%	No.	%	
Several days	42	100%	42	87.5%	0.028
One week	0	0%	6	12.5%	

* Fisher’s exact test; No.—number.

**Table 4:** Distribution of the patients according to gender, environment of origin, and analgesic usage.

**Gender**					
**Analgesic usage**	Female		Male		*p* *
	No.	%	No.	%	
Absent	38	36.5%	48	53.3%	0.021
Present	66	63.5%	42	46.7%	
**Living environment**					
**Analgesic usage**	Rural		Urban		*p* *
	No.	%	No.	%	
Absent	20	34.5%	66	48.5%	0.083
Present	38	65.5%	70	51.5%	
**Living environment—Female respondents**					
**Analgesic usage**	Rural		Urban		*p* *
	No.	%	No.	%	
Absent	2	12.5%	36	40.9%	0.046
Present	14	87.5%	52	59.1%	
**Living environment—Male respondents**					
**Analgesic usage**	Rural		Urban		*p* *
	No.	%	No.	%	
Absent	18	42.9%	30	62.5%	0.090
Present	24	57.1%	18	37.5%	

* Fisher’s exact test; No.—number.

**Table 5:** Distribution of the patients according to gender, diet impairment, and analgesic usage.

**Diet impairment**					
**Analgesic usage**	No DI		DI		*p* *
	No.	%	No.	%	
No analgesic usage	56	50.9%	30	35.7%	0.041
With analgesic usage	54	49.1%	54	64.3%	
**Diet impairment—Female respondents**					
**Analgesic usage**	No DI		DI		*p* *
	No.	%	No.	%	
No analgesic usage	26	52%	12	22.2%	0.002
With analgesic usage	24	48%	42	77.8%	
**Diet impairment—Male respondents**					
**Analgesic usage**	No DI		DI		*p* *
	No.	%	No.	%	
No analgesic usage	30	50%	18	60%	0.502
With analgesic usage	30	50%	12	40%	

* Fisher’s exact test; No.—number; DI—diet impairment.

## Data Availability

The data presented in this study are available on request from the corresponding authors. The data are not publicly available due to privacy reasons.
